# The Role of Neutrophil Extracellular Traps in Lipopolysaccharide-Induced Depression-like Behaviors in Mice

**DOI:** 10.3390/brainsci11111514

**Published:** 2021-11-15

**Authors:** Yue Kong, Guiqin He, Xiaolin Zhang, Jin Li

**Affiliations:** 1 The Key Laboratory of Developmental Genes and Human Disease, School of Life Sciences and Technology, Southeast University, Nanjing 210018, China; 2 Shanghai Key Laboratory of Psychotic Disorders, Shanghai Mental Health Center, Shanghai Jiao Tong University School of Medicine, Shanghai 200025, China; heguiqin1990@126.com (G.H.); LLL_smc@163.com (X.Z.); 3 Shanghai Clinical Research Center for Mental Health, Shanghai 200032, China

**Keywords:** neutrophil extracellular traps, inflammation, depression-like behavior, microglia

## Abstract

Peripheral inflammation plays a key role in the development of depression-like behaviors. However, the mechanisms underlying these effects remain largely unknown. Here, we found that the level of citrullinated histone H3 (cit-H3) significantly increased in the plasma of wildtype mice treated with lipopolysaccharide (LPS), which indicated that neutrophil extracellular traps (NETs) were formed. Moreover, the LPS-induced depression-like and asocial behaviors were significantly alleviated in the mice deficient of NETs. Mechanistically, NETs formation aggravated peripheral inflammation by increasing the concentrations of TNF-α, IL-1β and IL-6 in plasma, which are major proinflammatory cytokines that can enter the brain, resulting in microglia activation and reduced astrocytes. Following this, increased TNF-α and IL-1β were released into brain, inducing neuroinflammation and finally depression-like behaviors. Prohibiting NETs by PAD4 ablation significantly prevented LPS-induced microglia activation and the loss of astrocytes. Our results propose the role for peripheral NETs in LPS-induced depression-like behavior, and that NETs might be a potential target to prevent inflammation-induced major depressive disorder.

## 1. Introduction

Major depressive disorder (MDD) ranks as the first cause of disability all over the world, and more than 300 million people suffer from this devastating disorder [1]. Even though multiple effective treatments for MDD are accessible, about one third of patients achieve little remission following treatment [2], contributing to the global disease burden. Hence, it is very important to explore the mechanisms underlying MDD and to enable the development of novel therapies.

There has been growing evidence that immune activation is closely associated with the pathophysiology of MDD. Multiple clinical studies have shown that MDD patients have more C-reactive protein (CRP), proinflammatory cytokines interleukin (IL)-1β, IL-6 and tumor necrosis factor (TNF)-α in the blood than the healthy controls [3,4,5]. A recent meta-analysis revealed increased IL-6, IL-10, TNF and soluble TNF receptor (sTNFR)2 in MDD patients [6]. Moreover, suicide victims with depression showed higher levels of IL-1β, IL-6 and TNF in postmortem brains, while the receptors for the production of these cytokines, such as Toll-like receptor 3 (TLR3) and TLR4, were also affected [7,8,9]. In addition, depression-like behaviors are recapitulated by the acute infection of lipopolysaccharide (LPS) [10], and these behaviors are eliminated by antidepressant treatment before the LPS exposure [11]. Overall, some depression patients undergo inflammation alteration and cytokines, which attract more and more attention and have been studied extensively, but little is known about the source of the cytokines in MDD, and how the cytokines contribute to MDD is not fully understood.

Neutrophils, the most abundant leukocytes in blood, are considered as the first line of innate immune defense. In addition to phagocytosis and degranulation, the release of neutrophil extracellular traps (NETs) is one effective strategy to clear bacteria during infection [12,13,14]. NETs are extracellular web-like structures that mainly consist of cytosolic and granule proteins such as neutrophil elastase, cathepsin G and myeloperoxidase (MPO) that are assembled on decondensed chromatin [15]. Recently, NETs have been reported as being associated with autoimmune disorders [16], inflammation [17], tumor metastasis [18], Alzheimer’s disease [19] and stroke [20]. In the early stages of various pathological processes, e.g., ischemic stroke and traumatic injury, neutrophils can infiltrate the damaged brain tissue [21,22,23]. Moreover, NETs have been implicated in diverse pathological conditions of the brain. The increased expression of citrullinated histone H3 (cit-H3), which represents the formation of NETs in neutrophils, has been observed in the brains of animal models, including cerebral ischemia and intracerebral hemorrhage [24,25]. However, it is unknown whether NETs are involved in MDD.

The aim of this study was to evaluate the induction of NETs in mice with depression-like behaviors induced by LPS, as well as the alterations in depressive-like behaviors following the blocking of NETs formation. In addition, we detected the proinflammatory cytokines after LPS administration in both the plasma and the brain of wildtype and PAD4 knockout mice, in which NETs cannot be formed because the PAD4 protein necessary for NETs formation is deleted. We also assessed the number of microglia and astrocytes that may underlie the neuroinflammation.

## 2. Materials and Methods

### 2.1. Animals

Two–three months old C57BL/6 male mice and PAD4 KO male mice were housed under a standard 12 h light/12 h dark cycle condition at the Experimental Animal Center at Southeast University, China. All animal experiment procedures were approved by the Southeast University Animal Care and Use Committee (20120926001).

### 2.2. Systemic LPS Administration

LPS was obtained from Sigma-Aldrich (Sigma-Aldrich, St Louis, MO, USA). The mice were treated i.p. with LPS (1 mg/kg) dissolved in sterile saline. Animals were subjected to several behavioral tests 24 h after LPS treatment. Then, the animals were deeply anesthetized with chloral hydrate and euthanized by decapitation. The brain tissues were rapidly removed and stored at −80 °C until assays.

### 2.3. Neutrophil Extraction and Immunostaining

C57BL/6 male mice were injected i.p. with LPS and saline, the blood samples were collected by eyeball extirpating after 24 h. Neutrophils were enriched using EasySep™ Mouse Neutrophil Enrichment Kit (STEMCELL) and cultured in poly-D-lysine pretreated 48-well plate. One hour later, the cells were fixed by 4% paraformaldehyde (PFA) at room temperature for 15 min, and then blocked with 5% FBS for 45 min. The cells were incubated at 4 °C overnight with primary antibodies against rabbit anti-MPO (1:500, Abcam, ab208670, Boston, MA, USA), followed by incubation with an Alexa Fluor 555-conjugated donkey anti-rabbit IgG secondary antibody (1:500, Invitrogen, A31572, Thermo Fisher Scientific, Waltham, MA, USA) at 37 °C for 120 min. DAPI (Southern Biotech, Birmingham, AL, USA) was used as nuclear stain. The images were collected under an inverted fluorescence microscope (BZ-X810, Keyence, Japan).

### 2.4. Immunohistochemistry (IHC)

C57BL/6 mice were deeply anesthetized and subjected to cardiac perfusion with saline followed by perfusion with pre-cooling 4% PFA. Brains were dissected and fixed with 4% PFA in PBS overnight at 4 °C and the PFA was replaced with 30% sucrose in PBS at 4 °C until the brains were saturated. Then, the brains were embedded in Tissue-Tek^®^ O.C.T. Compound and frozen by liquid nitrogen. The brains were cut into coronal sections of 25 μm using a Leica CM 1950 cryostat. Brain sections were washed in PBS for 10 min, permeabilized with 0.3% Triton X-100 in PBS and blocked with PBS containing 0.2% Triton X-100 and 10% FBS for 2 h at room temperature. The sections were then incubated with primary antibodies overnight at 4 °C, washed three times in 0.3% PBT and incubated in appropriate secondary antibodies for 2 h at 37 °C. After washing with 0.1 M PBS, coverslips were counterstained with DAPI. The primary antibodies used included: rabbit anti-Iba1 (Abcam, 1:500, ab18847); rabbit anti-GFAP (Cell Signaling Technology, 1:500,12389T). The secondary antibodies used was Alexa Fluor 555 donkey anti-rabbit IgG (Invitrogen, 1:500, A31572). Immunostaining images were collected using BZ-X810 (Keyence, Japan).

### 2.5. ELISA

Proinflammatory cytokines and markers of NETs were determined by ELISA. The blood samples were collected by eyeball extirpating into the anticoagulant tube treated with EDTA. Then centrifuged at 3000 rpm for 15 min at 4 °C to generate plasma samples. The whole brain was removed after PBS perfusion via heart under anesthesia. The cortex and hippocampus were dissected and homogenized in phosphate buffered saline (PBS) buffer with protease and phosphatase inhibitors (Thermo Fisher) and centrifuged (12,000 rpm, 4 °C, 5 min). The supernatant was collected for ELISA. All samples were immediately stored at −80 °C until assay. The assay was performed according to ELISA Kit protocols of cit-H3 (Cayman, 501,620) and TNF-α, and IL1-β and IL-6 (MULTI SCIENCE, Hangzhou, China). A microplate reader (Varios Kan Flash, Thermo Fisher Scientific, Waltham, MA, USA) was used to measure absorbance at 450 nm and 570 nm. The levels of cytokines in plasma were expressed in pg/mL and the levels of protein in the brain were expressed in ng/mL.

### 2.6. Quantitative RT–PCR

RNA of tissues (hippocampus and cortex) was extracted with Trizol (Sangon Biotech, Shanghai, China). Total RNA quantity was measured using a BioPhotometer (Eppendorf, Hamburg, Germany). Reverse transcription from 2 µg of RNA was performed using the Hiscript Ⅱ 1st Strand cDNA Synthesis Kit (Vazyme, Nanjing, China). Real-time PCR was performed using Power Syber Green PCR Master Mix (Vazyme, Nanjing, China) and detected by the LightCycler Instrument (Roche Diagnostics, Basel, Switzerland) with software version 1.5.1.62. Results were normalized to the expression of the housekeeping gene Actin. The cycling parameters were as follows: stage 1, 95 °C for 5 min; stage 2, 40 cycles of 95 °C for 10 s and 60 °C for 30 s; stage 3, 95 °C for 15 s, 60 °C for 60 s and 95 °C for 15 s, which were concluded by the melting curve analysis process, and fold changes of gene expression were calculated using the 2−ΔΔCt method. The sequences used in this analysis were as follows: Tnf: forward 5′-CACGCTCTTCTGTCTACTGAACTTC-3′, reverse 5′-GCAGCCTTGTCCCTTGAAGAGAACC-3′; Il1b: forward 5′-GCAACTGTTCCTGAACTC-3′, reverse 5′-CTCGGAGCCTGTAGTGCA-3′; Actb: forward 5′-CATCCGTAAAGACCTCTATGCC-3′, reverse 5′-GACTCATCGTACTCCTGCTTG-3′.

### 2.7. Behavioral Assay

C57BL/6 mice were housed two–three per cage in a 12 h light/12 h dark cycle. All behavioral tests were performed during the light cycle. Prior to tests, mice were recovered for a week and handled for 5 min a day for 3 days. Mice were subjected to behavioral tests after 24 h of LPS injection. The tests were conducted, in order, as the open field test (OFT), forced swim test (FST) and tail suspension test (TST).

#### 2.7.1. Open Field Test (OFT)

The open field apparatus was a rectangular Plexiglas box (50 × 50 × 50 cm) comprising four walls and an open roof. The floor divided into 25 squares. Nine squares were defined as the center and the 16 squares along the walls as the periphery. Each mouse was tested for 10 min. Each subject was put in the same place of the apparatus’ arena near the center and allowed to explore the apparatus for 10 min. The apparatus was cleaned thoroughly with 75% ethanol before each subject was tested. The movement of the mouse was video tracked and analyzed off-line using VisualTrack (Xin Run, China).

#### 2.7.2. Forced Swim Test (FST)

The mice were placed in transparent cylindrical (30 cm in height, 12 cm in diameter) barrels two-thirds filled with tap water (23 ± 1 °C). After 1 min habituation, the immobility time of the mice was recorded for a further 5 min. The immobility times were recorded and analyzed by the TopScan video tracking software.

#### 2.7.3. Tail Suspension Test (TST)

Hanging the mice upside down, 40 cm above the ground, by taping them 1 cm from the tip of their tails. During a 6-min test period, the immobility time was scored for the last 5 min following a 1-min habituation by the TopScan video tracking software. In addition, the time from the beginning of the video to the first immobility of the mouse was measured as the latency to the first immobility.

### 2.8. Statistical Analysis

All analyses were carried out with GraphPad 8.0 software (San Diego, CA, USA). Data are shown as the mean ± SEM. The significance of the difference between the two groups was identified using a Student’s *t*-test. Multiple comparisons were performed by two-way ANOVA with Tukey post hoc test for comparisons among groups. All the mice used for experimentation and analysis were randomly allocated into groups. Random allocation was maintained throughout the study. A *p*-value < 0.05 indicated statistical significance. Significant differences were as follows: * *p*  <  0.05; ** *p*  <  0.01; *** *p*  < 0.001; **** *p*  <  0.0001.

## 3. Results

### 3.1. NETs Formation Is Induced in Mice with Depression-like Behaviors

**Figure 1:**
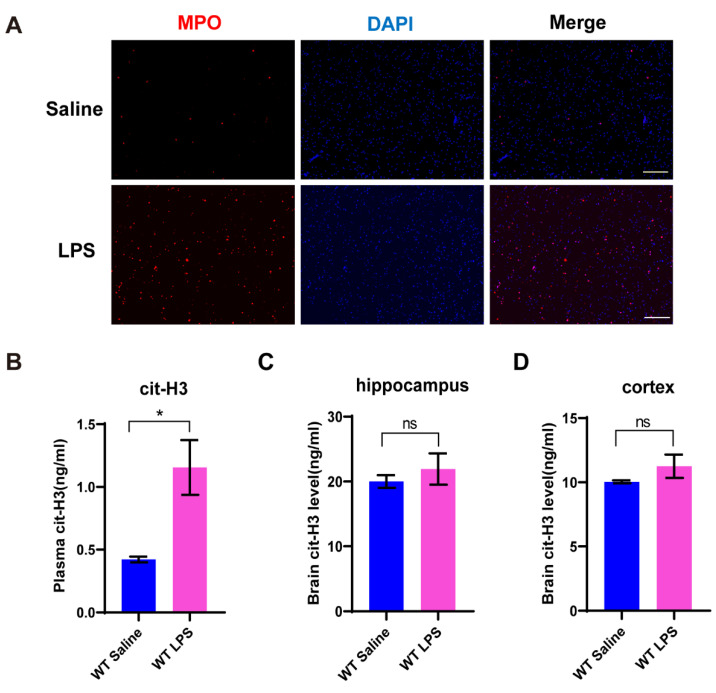
LPS administration intraperitoneally does indeed induce NETs in peripheral blood. (**A**) Neutrophils were extracted from peripheral blood, in mice that were injected with saline and LPS, respectively. Myeloperoxidase (MPO) is one marker of NETs. (**B**) cit-H3 is another marker of NETs. The level of cit-H3 in plasma was measured by ELISA. (**C**,**D**) Brain tissues were separated into hippocampus and cortex, then tested the level of cit-H3 in brain homogenate by ELISA. Data were expressed mean ± SEM. * *p* < 0.05 as compared to saline-treated control. *n* = 4 for ELISA test; *n* = 3 for immunostaining.

To verify whether NETs formation was induced during the development of depression, we treated mice with 1 mg/kg LPS intraperitoneally and took the eyeball for collecting peripheral blood after 24 h. Neutrophils were extracted from the peripheral blood and stained with myeloperoxidase (MPO), which was presented in NETs. As shown, the expression of MPO in the wildtype (WT) mice challenged with LPS was significantly upregulated compared to the saline group (Figure 1A). Furthermore, the ELISA data indicated that the level of cit-H3 in the peripheral blood obviously increased after LPS exposure (Figure 1B). However, the level of cit-H3 in brain homogenate of the hippocampus and the cortex had no significant change after LPS injection in the WT mice (Figure 1C,D). These data suggested NETs formation was induced in the peripheral blood, but not in the brain, during the development of the depression-like behaviors.

### 3.2. Depression-like Behaviors Are Relieved in PAD4 KO Mice

**Figure 2:**
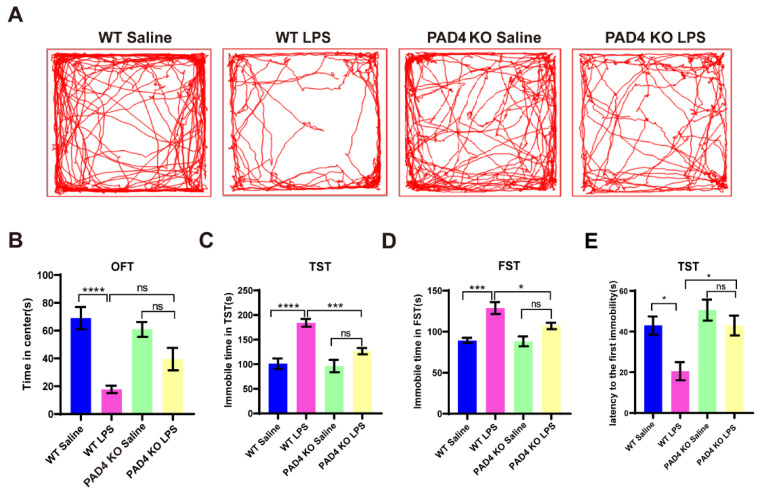
PAD4 knockout alleviated depression-like behaviors in LPS-treated mice. (**A**) Total distance traveled and line crossing in the open field. (**B**) Total time spent in the center of the open field. (**C**) Immobile time during the TST. (**D**) Immobile time during the FST. (**E**) The latency to the first immobility in TST. Data were expressed mean ± SEM (*n* = 9). * *p* < 0.05, *** *p* < 0.001, **** *p* < 0.0001. WT Saline: *n* = 7; WT LPS: *n* = 8; PAD4 KO Saline: *n* = 7; PAD4 KO LPS: *n* = 9.

To investigate the potential role of NETs in the development of LPS-induced depression-like behaviors, we used PAD4 KO mice in which NETs formation was blocked. We treated the mice with 1 mg/kg LPS i.p. and assessed the depression-like behaviors 24 h after LPS exposure. In the OFT, the total traveling distance and staying time in the center were obviously reduced in the WT LPS mice when compared to the WT saline group, but they did not change significantly in the PAD4 KO mice (Figure 2A,B). Meanwhile, in both TST and FST, the immobility time markedly increased in the WT mice after LPS injection, which did not change significantly in the PAD4 KO mice. Moreover, the immobility time in the PAD4 KO LPS group was significantly less than the WT LPS group (Figure 2C,D). We also detected the latency to the first immobility in TST. After the LPS treatment, the WT mice spent less time to give up a struggle, but in the PAD4 KO mice, they showed no significant change (Figure 2E). All the results of the behavioral experiments indicated that NETs contributed to the LPS-induced depression-like behaviors.

### 3.3. Proinflammatory Cytokines in Plasma Is Decreased in PAD4 KO Mice

**Figure 3:**
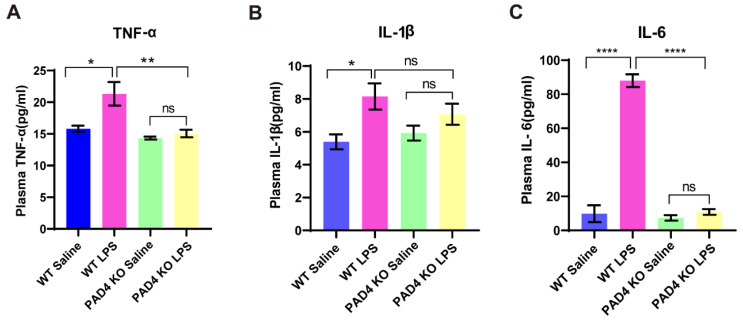
The level of plasma proinflammatory cytokines after LPS treatment. (**A**–**C**) The levels of plasma TNF-α, IL-1β and IL-6. Data were expressed mean ± SEM. * *p* < 0.05, ** *p* < 0.01, **** *p* < 0.0001. WT Saline, PAD4 KO Saline, PAD 4 KO LPS: *n* = 6; WT LPS: *n* = 9.

We then explored the mechanisms associated with the alleviated depression-like behaviors in PAD4 KO mice. Given the essential role of NETs in innate immunity, we detected the levels of inflammatory cytokines in the plasma of mice after LPS administration. We found the levels of TNF-α, IL-1β and IL-6 were significantly increased in the WT mice treated with LPS, which did not change significantly in the PAD4 KO mice (Figure 3). In addition, the levels of TNF-α and IL-6 in the PAD4 KO LPS mice were obviously lower compared to the WT LPS mice (Figure 3A,C). These data illustrated that NETs may promote inflammation in the periphery by increasing the release of TNF-α, IL-1β and IL-6.

### 3.4. Proinflammatory Cytokines in Brain Is Reduced in PAD4 KO Mice

**Figure 4:**
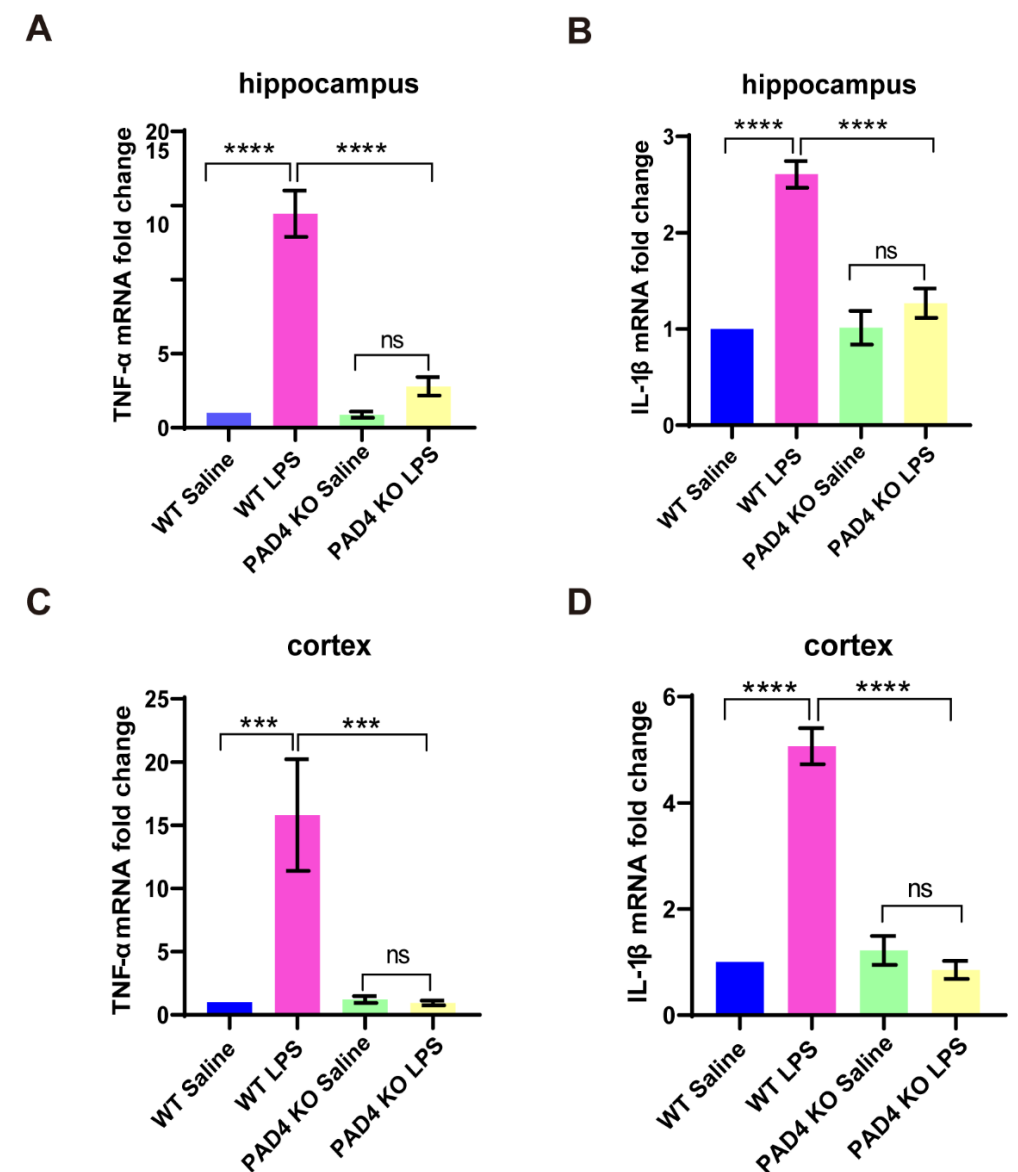
PAD4 knockout attenuates the neuroinflammation induced by LPS. (**A**,**B**) The mRNA level of TNF-α and IL-1β in hippocampus after LPS or saline treatment. (**C**,**D**). The mRNA level of TNF-α and IL-1β in cortex after LPS or saline treatment. Data were expressed mean ± SEM. *** *p* < 0.001, **** *p* < 0.0001. *n* = 3. WT Saline: *n* = 4; WT LPS: *n* = 4; PAD4 KO Saline: *n* = 3; PAD4 KO LPS: *n* = 5.

Neuroinflammation is tightly associated with the onset of depression [26,27]. Next, to confirm whether the protective effects of PAD4 knockout in the pathological process were exerted by mediating the neuroinflammation, we extracted the total RNA of the hippocampus and the cortex from both the WT and PAD4 KO mice. The RT-PCR data demonstrated that the levels of TNF-α and IL-1β were significantly increased in both the hippocampus and the cortex after LPS administration in the WT mice, but in the PAD4 KO mice, these cytokines had no significant change. In addition, the levels of TNF-α and IL-1β in the PAD4 KO LPS group were significantly lower than the WT LPS group (Figure 4A–D). The results suggested that blocking NETs can attenuate the neuroinflammation induced by LPS.

### 3.5. The Influence on Glia Cells Can Be Relieved in PAD4 KO Mice

**Figure 5:**
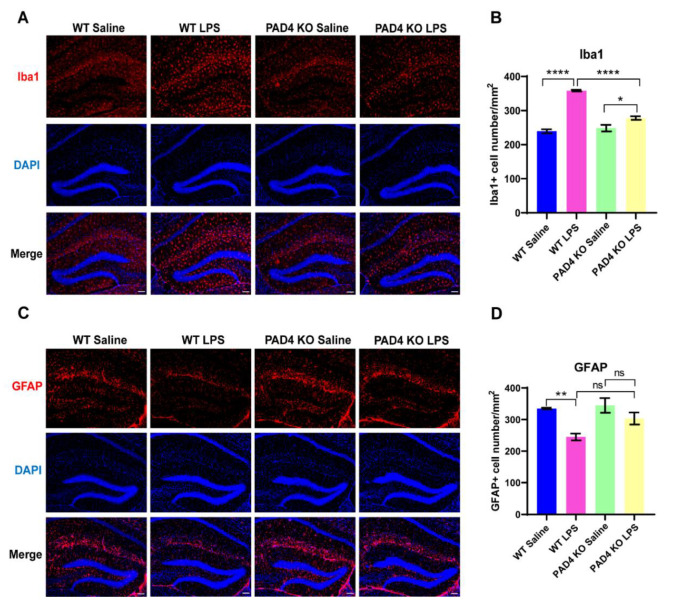
The influence of LPS on glia cells among the PAD4 KO and wildtype mice. (**A**) Representative immunofluorescence of Iba-1 in the hippocampus. (**B**) Column graphs representing Iba-1 expression. (**C**) Representative immunofluorescence of GFAP in the hippocampus. (**D**) Column graphs representing GFAP expression. Data were expressed mean ± SEM. * *p* < 0.05, ** *p* < 0.01, **** *p* < 0.0001. bar = 100 um; WT Saline, WT LPS, PAD4 KO Saline: *n* = 3; PAD4 KO LPS: *n* = 4.

Activated glial cells, including microglia and astrocytes, are key sources of neuroinflammation and central cytokines [28,29,30]. To examine the involvement of microglia and astrocytes in LPS-induced neuroinflammation, the expression of the glial cell activation markers’ ionized calcium-binding adaptor molecule (Iba-1) and glial fibrillary acidic protein (GFAP) was determined by immunofluorescence. After LPS injection, Iba1 was upregulated significantly in both the WT and PAD4 KO mice, but compared to the WT LPS mice, Iba1 expression was markedly lower in the PAD4 KO LPS mice (Figure 5A,B). The expression of GFAP was decreased significantly in the WT mice after LPS treatment, but in the PAD4 KO mice, the expression of GFAP did not change between the saline and LPS groups (Figure 5C,D). Altogether, these results suggested that NETs promoted neuroinflammation by microglia activation and astrocytes reduction.

**Figure 6:**
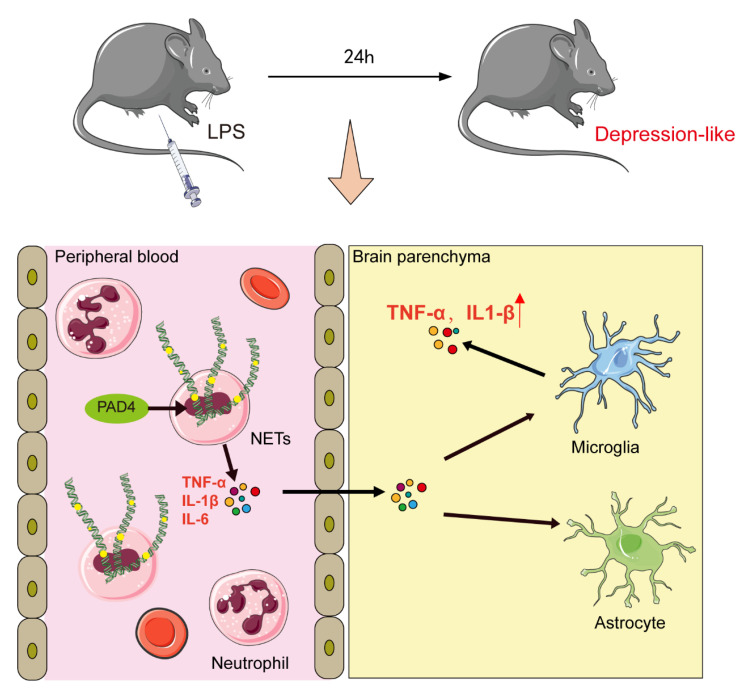
Mechanism diagram of NETs in LPS-induced depression-like behaviors.

Finally, we summarized the whole study and put forward an assumption that will explain the mechanism of NETs in LPS-induced depression-like behaviors (Figure 6). We observed the following findings: (1) NETs formation was significantly increased in mice after LPS administration; (2) NETs formation aggravated TNF-α, IL-1β and IL-6 release into peripheral blood; (3) these cytokines can cross the BBB, leading to microglia activation and astrocytes reduction, which induced neuroinflammation and further depression-like behaviors.

## 4. Discussion

Inflammation and cytokines have been previously reported in depressed patients and mice [31], but the source of the cytokines and how they contribute to depression is not completely known. Here, we demonstrated first that NETs formation was induced in the peripheral blood of mice with depression-like behaviors, and the deletion of NETs in PAD4 KO mice relieved the depression-like behaviors. Further, we observed increased proinflammatory cytokines in both the peripheral blood and the brain, which decreased in the PAD4 KO mice without NETs. Finally, we showed microglia activation after LPS treatment, and this was reduced with the deletion of NETs.

The injection of LPS induced depressive-like behavior, which is an already validated model of inflammation-induced depression. In this study, we observed NETs formation in isolated neutrophils from mice with depression-like behaviors after 24 h of 1 mg/kg LPS treatment. A recent study also showed isolated neutrophils from C57BL/6 mice underwent obvious NETs formation after 6 h of 10 µg LPS was given intraperitoneally [32]. Moreover, we investigated NETs not induced in the brain. There is a possibility that the brain–blood barrier (BBB) integrity was not disturbed by LPS, and no neutrophils were attracted to the brain. Brain-transmigrated neutrophils were increased 24 and 48 h after 2.5 mg/kg LPS was administrated intraperitoneally [33]. The BBB was damaged after 72 h of 1 mg/kg LPS or 24 h after 5 mg/kg LPS was given intraperitoneally [34,35]. In our animal model, 1 mg/kg LPS may not be able to impair the BBB and recruit neutrophils into the brain.

To date, there is no evidence that NETs play a role in the development of depression-like behaviors in response to LPS. We first demonstrated the depression-like behaviors were abrogated in mice without NETs. Previous studies have reported LPS-induced systemic inflammation triggered by peripheral neutrophils recruitment into the brain parenchyma, and these cells are essential factors for depression-like behaviors [33]. Indeed, depression cases had more neutrophils in the peripheral blood than in healthy controls, and the genes associated with neutrophil functions were upregulated or abnormally expressed [36,37,38]. However, whether NETs were triggered in the peripheral blood and played a role in the development of depression was not clear. We presented evidence that the peripheral neutrophils released NETs and contributed to the development of LPS-induced depression-like behaviors in mice. More proof was needed to confirm this in other depression models. 

In this study, we also revealed increased proinflammatory cytokines TNF-α, IL-1β and IL-6 after LPS treatment both in the peripheral blood and the brain, which was reduced in absence of NETs, indicating that part of the cytokines results from NETs formation. NETs are thought to clear bacteria, fungi and viruses [13,39,40]. However, NETs can also promote the inflammation activation and pathogenesis of diseases in conditions of excess. NETs amplify inflammation and liver damage in mice of ischemia-reperfusion injury, which can be significantly rescued in the mice treated with PAD4 inhibitors or DNase [41]. NETs turn on the transcription of IL-6 and pro-IL-1β genes in macrophages during the early inflammatory stages of atherosclerosis. The increased cytokines augments T helper 17 cell differentiation and recruits more myeloid cells to atherosclerotic lesions [42]. In addition, NETs formation was followed by a population of infiltrating monocytes, which are a major source of the inflammatory cytokines IL-6 and TNF-α, and NETs inhibition reduces the monocyte infiltration and inflammation [43]. Therefore, we speculated that the NETs formation of circulating neutrophils in peripheral blood might aggravate the inflammation and depression-like behaviors.

The hippocampus and the cortex are the brain regions responsible for mood disorders such as depression, and cytokines in these regions are key factors in the pathophysiology of depression. We detected significantly higher levels of TNF-α and IL-1β in the hippocampus and the cortex of mice exposed to LPS, indicating that neuroinflammation was activated. Similar results were observed in other animal models of depression [44,45,46]. Toll-like receptor 4 (TLR4) is the primary receptor for the production of cytokines induced by LPS, and the expression of TLR4 in the hippocampus is upregulated in mice with depression-like behaviors accompanying neuroinflammation [44,47].

Multiple studies have suggested that adult hippocampal neurogenesis plays a vital role in the process of depression based on the finding that adult hippocampal neurogenesis is reduced both in MDD patients and in depression animal models [48,49,50]. Our results have demonstrated increased proinflammation cytokines in the hippocampus, such as TNF-α and IL-1β, which all contribute to decreased adult hippocampal neurogenesis [51,52]. In rats, the administration of IL-1β led to adult hippocampal neurogenesis reduction, which also promotes depression-like behaviors [53]. So, increased TNF-α and IL-1β in the hippocampus in our work may contribute to depression-like behaviors by affecting adult hippocampal neurogenesis.

In our work, we observed obviously decreased proinflammatory cytokines in the periphery in mice with deficient NETs. Meanwhile, neuroinflammation was also relieved. It is possible that peripheral cytokines promote inflammation in the brain through a specific pathway, which then leads to depression. Numerous studies have elucidated that inflammation signals in the periphery can be conveyed to the brain, which drives inflammation response in the brain and results in the development of depression. Cytokines, e.g., TNF-α, IL-1β and IL-6, have a direct effect on microglia, astrocytes and neurons, which are major sources of cytokines in brain, after entering the brain by saturable transport [54]. The receptors on the cell surface of the brain’s endothelial cells and astrocytes composing the BBB are involved in the brain activation by circulating cytokines. Moreover, TNF-α and IL-1β may bind with the peripheral afferent nerve fibers, which in turn stimulate ascending catecholaminergic fibers in the brain to affect depression.

Microglia activation and astrocytes reduction were obvious in our animal model, and both were rescued in mice with deficient NETs, which indicated that NETs affected the depression-like behaviors by aggravating neuroinflammation induced by increased microglia activity. A recent human study shows that more microglia activation is detected in cortical areas that have direct correlation with depression severity [55]. Another meta-analysis suggests that increased microglial activity is associated with increased TNF levels in the brain parenchyma of MDD patients [56] and reduced astrocytes numbers in MDD patients [57]. There is another point that the reduction of astrocytes may mean a more permeable BBB, allowing the penetration of peripheral substances directly into the brain, attracting monocytes to the brain parenchyma [57].

## 5. Conclusions

In summary, we have identified a novel mechanism that regulates the development of depression-like behaviors during acute peripheral inflammation. This involves the NETs formation in peripheral blood, leading to increased proinflammatory cytokines, which may cross the BBB and activate microglia in brain. Following this, neuroinflammation was triggered and finally led to depression-like behaviors. These results suggested that NETs may represent a potential target for inflammation-induced depression.

## Data Availability

The data presented in this study are available on request from the corresponding authors.
